# Supplementary material for: Integrating genomic structural equation modeling and experimental validation to unravel the genetic basis of male genital lichen sclerosus
Source: Front Immunol. 2026 Jul 16;17:1857945. doi: 10.3389/fimmu.2026.1857945 (PMC13435250; doi:10.3389/fimmu.2026.1857945)
Supplement: Supplementary file 6 [file SupplementaryFile5.zip › result/fuma_PSYCH_factor_sCCA1_focus.chr12.chr12.61107.1080331.pop1.pdf]

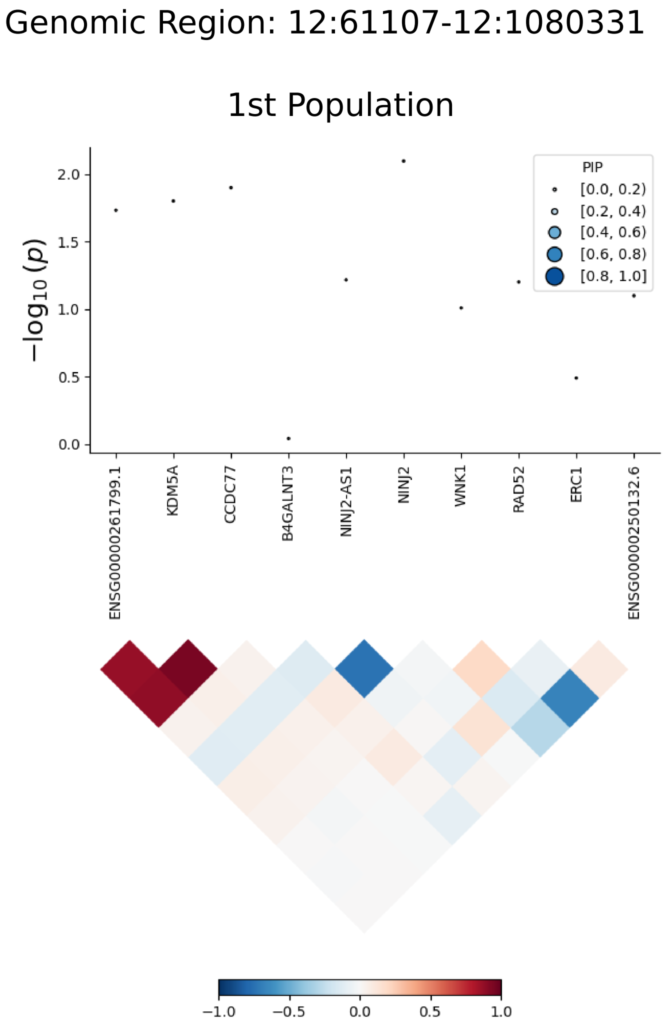

Genomic Region: 12:61107-12:1080331
1st Population
−log10 (p)
2.0
1.5
1.0
0.5
0.0
PIP
[0.0, 0.2)
[0.2, 0.4)
[0.4, 0.6)
[0.6, 0.8)
[0.8, 1.0]
ENSG00000261799.1
KDM5A
CCDC77
B4GALNT3
NINJ2-AS1
NINJ2
WNK1
RAD52
ERC1
ENSG00000250132.6
−1.0
−0.5
0.0
0.5
1.0
